# Antimicrobial Mixture Based on Micronized Kaolinite and Ziziphora Essential Oil as a Promising Formulation for the Management of Infected Wounds

**DOI:** 10.3390/ijms252313192

**Published:** 2024-12-08

**Authors:** Aigerim A. Karaubayeva, Tolkyn Bekezhanova, Karlygash Zhaparkulova, Katarzyna Susniak, Jan Sobczynski, Paulina Kazimierczak, Agata Przekora, Krystyna Skalicka-Wozniak, Łukasz Kulinowski, Anna Glowniak-Lipa, Zuryiadda B. Sakipova, Izabela Korona-Głowniak

**Affiliations:** 1 School of Pharmacy, Asfendiyarov Kazakh National Medical University, Almaty 050000, Kazakhstan; karaubayeva.a@kaznmu.kz (A.A.K.); bebezhanova.t@kaznmu.kz (T.B.); zhaparkulova.k@kaznmu.kz (K.Z.); sakipova.z@kaznmu.kz (Z.B.S.); 2 Department of Pharmaceutical Microbiology, Medical University of Lublin, 20-093 Lublin, Poland; katarzyna.susniak@umlub.pl; 3 Department of Clinical Pharmacy and Pharmaceutical Care, Medical University of Lublin, Chodźki St. 7, 20-093 Lublin, Poland; jan.sobczynski@umlub.pl; 4 Department of Tissue Engineering and Regenerative Medicine, Medical University of Lublin, 20-093 Lublin, Poland; paulina.kazimierczak@umlub.pl (P.K.); agata.przekora@umlub.pl (A.P.); 5 Department of Natural Products Chemistry, Medical University of Lublin, 20-093 Lublin, Poland; kskalicka@pharmacognosy.org (K.S.-W.); lukasz.kulinowski@umlub.pl (Ł.K.); 6 Department of Cosmetology, University of Information Technology and Management in Rzeszów, Sucharskiego 2, 35-225 Rzeszow, Poland; aglowniak@wsiz.edu.pl

**Keywords:** kaolin, antimicrobial activity, collagen production, skin infections

## Abstract

Kaolinite stands out as a promising natural geomaterial for developing new therapeutic systems aimed at addressing global health challenges, such as multidrug-resistant infections. In this study, we report on the formulation and biological activity of a therapeutic mixture composed of white micronized kaolinite (KAO) and Ziziphora essential oil (ZEO), intended for topical application on infected wounds. GC–MS analysis revealed that the primary component of ZEO is pulegone, constituting 72.98% of the oil. ZEO demonstrated good bioactivity against bacterial and fungal strains (MIC 1.25–5 mg/mL). Additionally, ZEO at a concentration of 0.0156% (0.156 mg/mL) was found to significantly stimulate collagen synthesis. The antimicrobial activity of the tested KAO–ZEO mixture formulation (30% KAO/0.25% ZEO in an excipient base) showed the highest effectiveness against *Candida* spp. (MIC 0.08–25 mg/mL) and Gram-positive bacteria (MIC 0.16–25 mg/mL), with lower activity against Gram-negative bacteria (MIC 25–50 mg/mL). Moreover, the KAO–ZEO mixture was nontoxic (cell viability near 100%) to human skin fibroblasts according to the ISO 10993-5 standard and promoted collagen synthesis by skin cells. This is the first documented formulation combining KAO and ZEO, demonstrating significant antimicrobial properties along with the ability to stimulate collagen production in fibroblasts. These properties highlight KAO–ZEO as a promising novel treatment, which may synergize with current care standards and improve wound healing outcomes.

## 1. Introduction

Kaolinite has been widely employed in various pharmaceutical applications both as an excipient and an active ingredient due to its outstanding physical, chemical, and surface physicochemical properties. Beyond its traditional roles in pharmaceuticals, kaolinite and its derivatives have recently gained attention as promising materials in innovative biomedical fields such as drug, protein, and gene delivery. This is attributed to their strong interactions with organic and biochemical molecules, bioadhesive capabilities, and potential for cellular uptake [1]. Due to its preserved bioactivity, kaolinite has also been utilized as an active agent in treating various common ailments. It can be applied topically as a hemostatic, dermatological protector, anti-inflammatory agent, or in therapeutic mud treatments (pelotherapy). When administered orally, it acts as a gastrointestinal protector and shows antibacterial, antiviral, detoxifying, and antidiarrheal properties [2,3]. With these premises, the future of kaolinite in healthcare uses is strongly interesting, especially in the development of pharmaceutical and cosmetic industries. Historically, natural clays, including kaolinite, halloysite, montmorillonite, beidellite, talc, sepiolite, and palygorskite, have been widely used for the treatment of wounds and to stop hemorrhaging [4]. Thus, it is not surprising that there are reports in the available literature on wound dressing materials containing clay. Some studies describe combination of different clays (montmorillonite, kaolinite, and halloysite) with chitosan, polyvinyl pyrrolidone (PVP), polyethylene terephthalate (PET), or cotton to produce a hemostatic bandage [5]. Li et al. fabricated a graphene–montmorillonite composite sponge with the ability to rapidly stop bleeding in approximately 85 s in rabbit artery injury test [6]. In turn, Long et al. developed bifunctional hybrid fiber membrane of ZnO–Fe_2_O_3_/kaolinite nanoclay/poly-(3-caprolactone)-gelatin with the ability to control bleeding, prevent bacterial colonization, reduce excessive inflammation, and facilitate wound healing in bacteria-infected mice models [7]. In this study, it was decided to develop kaolinite-based gel for the treatment of infected wounds, whose antimicrobial activity and pro-healing properties will be boosted due to enrichment of the formulation with *Ziziphora* essential oil.

Species of the genus *Ziziphora* (Lamiaceae) have long been utilized in traditional medicine for their sedative, antiseptic, carminative, and expectorant properties. *Z. clinopodioides* subsp. bungeana, is widely used in Kazakh folk medicine in the form of infusions, alcohol-based tinctures, and decoctions. These preparations serve as sedatives, expectorants, and carminatives, and are used to treat ailments like colds, inflammation, coughs, migraines, fever, diarrhea, and depression. In addition, aqueous tinctures are applied for symptoms associated with cardiovascular conditions such as coronary heart disease and hypertension, scrofula, and colds, as well as externally for rheumatism and toothache [8,9,10]. *Z. clinopodioides* subsp. *bungeana* is rich in diversity of bioactive compounds found in the plant, which include oleanolic and ursolic acids, phenolic compounds, flavonoids (pigenin, chrysin, linarin, and acacetin). Menthone (6.7%), isomenthone (28%), pulegone (55.2%), and thymol (2.5%) have been previously isolated from aerial parts of the plants as components of essential oil [11].

Mechanical injuries like cuts or burns can disrupt the skin’s protective barrier, exposing the underlying tissue to the external environment. This creates an ideal condition—warm, moist, and nutrient-rich—that fosters the growth of harmful microorganisms. As a result, the wound becomes susceptible to microbial colonization and infection. Wound infections not only worsen the patient’s trauma but also place a significant financial burden on the healthcare system [12]. For instance, many postoperative patients suffer from surgical site infections, which are linked to high morbidity and mortality rates. Approximately 25% of these patients develop severe sepsis, requiring admission to intensive care units [13]. The situation is further complicated by the rising occurrence of infections caused by multidrug-resistant (MDR) bacteria. According to the World Health Organization, over 2 million cases of illness are linked to MDR bacteria, with direct and indirect costs surpassing USD 55 billion annually [14].

Given the fact that kaolinite can be considered as a promising natural geomaterial for designing new derivatives contributing to the discovering new therapeutic systems, specifically topical, and treatment pathways of global challenge diseases, e.g., MDR infections, especially in skin infections, here, we report the composition of micronized kaolinite–ziziphora essential oil therapeutic mixture (KAO–ZEO) and its biological activity accounted for topical usage.

## 2. Results and Discussion

### 2.1. Characterization of Ziziphora Essential Oil—Bioactive Componenet of KAO–ZEO Gel Formulation

Kazakhstan holds natural resources of both plant and mineral origin that, when utilized strategically, can play a significant role in advancing the domestic pharmaceutical industry. The Alekseevskoye clay mineral deposit, located in the Akmola region of Kazakhstan, has extensive reserves of kaolinite clay (exceeding 260 million tons), ranking it fifth worldwide in terms of mineral resources. This deposit lies between the villages of Vasilkovka and Birlistik, approximately 27 km northwest of Kokshetau, with convenient transportation access via the Kokshetau–Petropavlovsk railway and the Kokshetau–Taiycha highway. The color and whiteness of the Alekseevskoye kaolins are influenced by iron-containing minerals like goethite, hematite, biotite, chlorite, sericite, and ilmenite. Titanium minerals present include rutile, ilmenite, and sericite [15].

The chemical composition, physicochemical properties, mineral composition, dispersibility, swelling and gel formation capacity, sorption characteristics, binding capacity, and safety evaluation for human and environmental quality of micronized kaolinite clay powder (KAO) from the Alekseevskoye deposit have previously been examined [16].

Research has shown that treating this clay with a hydrochloric solution of sodium hydrosulfite, in the presence of a complexing agent (such as oxalic acid or the disodium salt of ethylenediaminetetraacetic acid), enhances whiteness by approximately 5 units, an important improvement for applications requiring visually appealing excipients. The increase in whiteness is primarily due to the dissolution of iron-containing minerals like goethite, while the whiteness level of treated kaolin is affected by sericite content. Sericite-enriched fractions demonstrate a particular weight content of Fe_2_O_3_ [17].

Due to their broad range of properties, including sorption activity, binding and disintegration abilities, smoothness, form-shaping capability, and inertness, kaolinite clays are highly valued in modern pharmaceutical technology. Selecting the right excipients for a given dosage form requires prioritizing safety, functionality for the intended technological purpose, and cost-effectiveness.

**Figure 1:**
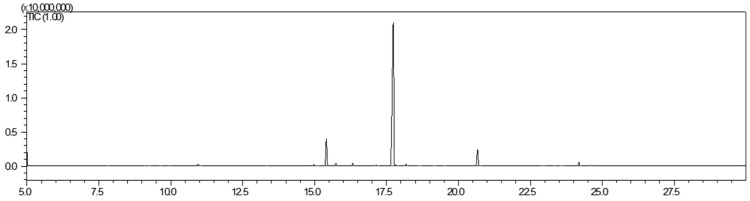
The GC–MS chromatogram of Ziziphora EO (ZEO).

GC–MS analysis was used to evaluate the compositions of *Z. bungeana* EO (ZEO) obtained via hydro-distillation constituted 1.6% of dry plant material. A total of 380 g of raw material was hydro-distilled. This yielded 6.08 mL of essential oil, which was colorless with an aromatic odor. The chemical composition of ZEO, including proposed identification (name), area (%), experimental retention indices (RI_exp_), databases retention indices (RI_db_), and retention times (RT) are presented in Table 1. The GC–MS chromatogram is presented in Figure 1.

The GC–MS analysis of the chemical composition of ZEO showed pulegone as the main ingredient with 72.98% content. A total of 18 constituents, representing 96.27% of the total oil, are presented in Table 1.

So far, Zhaparkulova et al. have identified eight components (representing 94.41% of the oil) in aerial parts of *Z. bungeana* Juz. (collected in the flowering stage at the foothills of the Dzungarian Alatau, Republic of Kazakhstan) such as pulegone (58.30%), isomenthone (13.49%), menthone (6.71%), α-limonene (5.79%), isopulegone (5.01%), isomenthol (2.63%), β-pinene (1.44%), and α-pinene (1.04%) [18]. Further work, but this time with extracts and application of LC-MS technology, enabled the identification of thymol, carvacrol, ziziphorosides isomers, possibly ziziphoroside A, B, and C [10].

Previous investigations of the chemical profile of essential oils from other *Ziziphora* species indicated pulegone as major compound in *Z. clinopodioides* and *Z. tenuior* [19,20,21,22]. Kazeminia et al. (2024) identified in the aerial parts of flowering *Ziziphora clinopodioides* L. pulegone (29.24%), 1,3-dimethyl-2-(2-methylpropylidene), imidazolidine (9.05%), piperitenone (6.65%), thymol (5.38%), and carvacrol (5.27%) as the most abundant components [19]. Pulegone (>55%), together with piperitenone (12.8%), and *iso*-menthone (8.0%) has consistently been reported as the predominant chemical compound in *Z. clinopodioides*, while pulegone (27.5%), borneol (16.5%), 1, 8-cineole (9.8%), camphor (8.37%), β-pinene (5.31%), and α-pinene (4.64%) were major compounds in *Z. tenuior*, which was described by other authors [20,21]. Pulegone (70.4%) together with menthone (11.5%) or pulegone (55.1%) and limonene (8.2%) were detected as major in essential oils from *Z. clinopodioides* and *Z. tenuior*, respectively [22].

### 2.2. Antimicrobial Activity of KAO, ZEO and CLEANED CLAY

We first evaluated the antimicrobial activity of two clay forms and ZEO against reference microorganisms to assess their potential for therapeutic mixture preparation. The antimicrobial assay results are summarized in Table 2. Tests with CLEANED CLAY showed lower antimicrobial activity than KAO, with MIC values > 20 mg/mL for Gram-positive bacteria, 10–20 mg/mL for Gram-negative bacteria, and similar MIC values for *Candida* spp. as KAO (5–10 mg/mL). Antimicrobial activity of KAO was observed as MIC values ranged from 5 to 10 mg/mL against tested both bacterial and fungal strains. Due to its safe bioactivity, kaolinite is being explored as an active agent in treatments for common diseases. Its antimicrobial properties stem from the gradual release of metal ions and its high surface charge, which enhances its affinity for proteins and cells to support tissue repair [23].

The bioactivity of ZEO (MIC values ranged from 1.25 mg/mL to 5 mg/mL against bacteria) was shown higher against fungal strains (MIC 1.25 to 2.5 mg/mL) and was consistent with previously reported activity for Ziziphora extracts [10]. ZEO showed moderate activity against both Gram-positive and Gram-negative bacteria, while earlier findings indicated Ziziphora extract was more active against Gram-positive bacteria [10]. Similar findings were reported by Sonboli et al. [11] for *Ziziphora clinopodioides* subsp. *bungeana* essential oil from Iran, with MIC values of 3.75 mg/mL for tested *Staphylococcus* spp. and *E. coli* strains [11]. Additionally, Ziziphora essential oil has shown satisfactory antimicrobial effects against foodborne pathogens, both alone and in combination with nisin, indicating its potential as a preservative [24].

### 2.3. Results of Formulation Assessment

**Figure 2:**
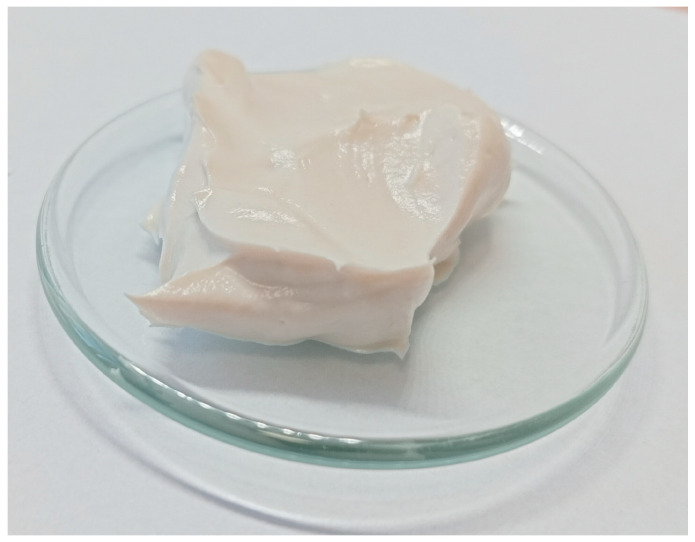
The picture of gel formulation containing micronized kaolinite clay and Ziziphora essential oil.

White micronized kaolinite clay powder (KAO) of the Alekseevskoye deposit in the Akmola region and Ziziphora essential oil (ZEO) were used to produce KAO–ZEO gel formulation. The quantity of several ingredients was selected based on antimicrobial activity of ZEO and KAO. A proper excipient selection ensured that active ingredients exhibited their activity. Glycerin and synthetic amorphous magnesium aluminometasilicate stabilized kaolinite clay dispersion in the gel matrix and thus precluded clumping. Polyglyceryl 4-caprate solubilized the Ziziphora essential oil and ensured its solubilization in aqueous vehicle. The produced gel was opaque, creamy white, smooth, with no visible lumps of kaolinite clay powder, slightly shiny formulation, as shown in Figure 2. Formulation was a low-viscosity dense gel with neutral pH. Formulation viscosity was evaluated to be 4.36 ± 0.13 Pa·s. Gel pH was equal to 7.46, whereas gel density was 1.196 ± 0.08 g/mL.

### 2.4. Antimicrobial Activity of ZEO–KAO Mixture

MIC assays of the prepared mixtures were performed for reference strains commonly associated with skin infections, including *S. aureus*, *S. epidermidis*, *E. faecalis*, *P. aeruginosa*, *E. coli*, *S. pyogenes*, and Candida spp. The tested formulation of KAO–ZEO mixture (30% KAO/0.25% ZEO in excipient base) as compared to formulations, each containing an individual ingredient in the excipient base, included the ZEO mixture (0.25% of ZEO in excipient base) and KAO mixture (30% kaolinite in excipient base). Their antimicrobial potential was evaluated against the selected pathogens, while excipients without active substances served as a negative control. MIC results are summarized in Table 3.

We observed that the antimicrobial activity of KAO–ZEO results primarily from the micronized kaolinite, which is the main active component of the mixture. ZEO is included at a concentration of 0.25% due to its cytotoxicity toward human skin fibroblasts. The antimicrobial effects of KAO–ZEO suggest its potential use in maintaining microbial balance and limiting the proliferation of bacteria and yeasts on the skin. It should be noted that antimicrobial activity of natural clays is well known in the available literature. It was proved that clay minerals possess antimicrobial activity due to the presence of metal complexes and cations in their structure capable of attracting toxins and absorbing pathogens, enhancing the healing of infected wounds. Moreover, the clay has also the ability to alter the pH, oxidation state, and osmotic strength of the surrounding environment catalyzing the bactericidal effects without significant loss in viability of eukaryotic cells [25,26].

### 2.5. Evaluation of the Cellular Response to ZEO and KAO–ZEO In Vitro

**Figure 3:**
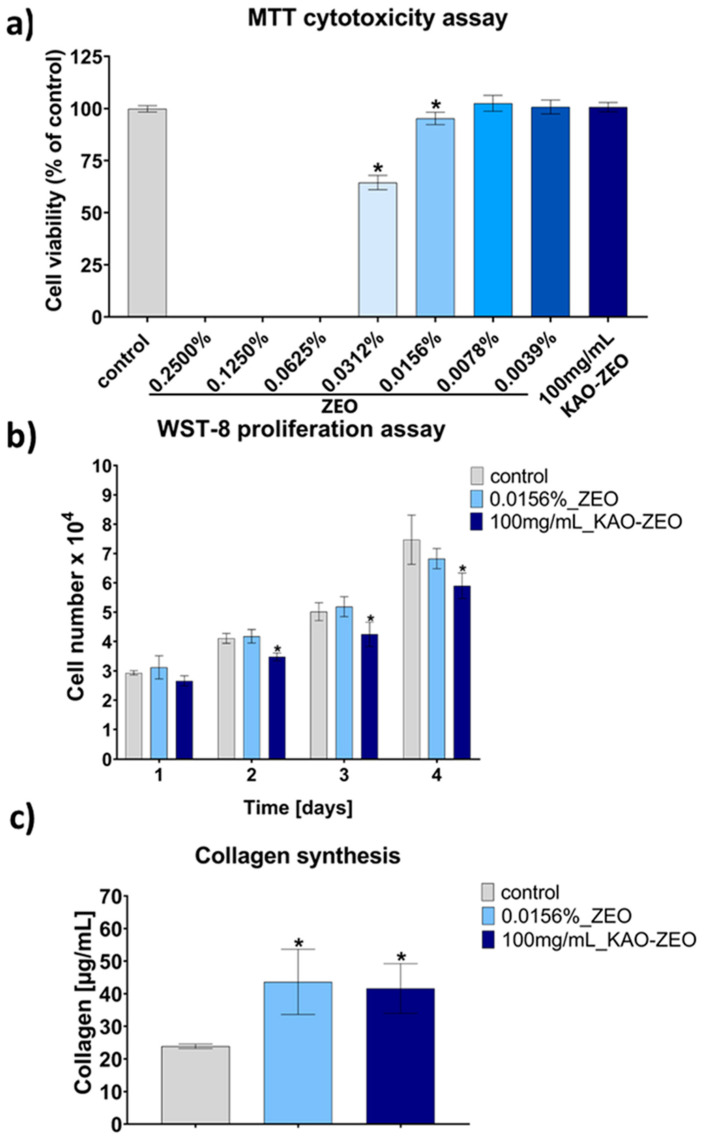
Evaluation of the biological properties of ZEO and KAO–ZEO on normal human skin fibroblasts (BJ): (**a**) Screening cytotoxicity test on different concentrations of ZEO ranging from 0.0039% to 0.2500% and cytotoxicity assessment of KAO–ZEO extract prepared by soaking 100 mg of the sample in 1 mL of the culture medium for 24 h at 37 °C; (**b**) Evaluation of cell proliferation after exposure to the highest non-toxic concentration of ZEO (0.0156%) and KAO–ZEO extract (prepared at the ratio 100 mg/mL); (**c**) Collagen synthesis assessment after exposure to the highest non-toxic concentration of ZEO (0.0156%) and KAO–ZEO extract (prepared at the ratio 100 mg/mL); (control—cells maintained in the culture medium without the ZEO and KAO–ZEO; * statistically significant results considered at *p* < 0.05 compared to the control cells according to One-way ANOVA with post hoc Dunnett’s test).

The in vitro biological properties of ZEO and KAO–ZEO were evaluated on normal human skin fibroblasts (BJ) using a cytotoxicity assay, followed by cell proliferation and collagen synthesis tests. Screening the cytotoxicity assay showed that ZEO at concentration range from 0.2500% to 0.0625% exhibited a strong cytotoxic effect towards BJ cells (no viable cells were detected), whereas ZEO at concentration of 0.0312% decreased cell viability to 64.5% (Figure 3a). ZEO at concentrations ranging from 0.0156% to 0.0039% did not exert cytotoxic effect towards BJ cells which exhibited high viability (above 95%). Therefore, the ZEO at a concentration of 0.0156% was selected as the highest non-toxic dose that was used in further experiments. Importantly, BJ fibroblasts exposed to the 24 h extract of the KAO–ZEO sample showed high viability (near 100%), proving nontoxicity and safety of this formulation (Figure 3a).

The increase in number of cells (proliferation) after exposure to ZEO at a concentration of 0.0156% and to KAO–ZEO extract was examined after 1, 2, 3, and 4 days of culture. Figure 3b shows that the proliferation potential of the cells after exposure to 0.0156% ZEO was similar to the control fibroblasts, suggesting that this treatment did not significantly hinder cell growth. In contrast, the number of BJ cells after 2, 3, and 4 days of culture in the presence of KAO–ZEO extract was significantly lower than the number of control cells, what may indicate a slight inhibition of cell proliferation. Importantly, the cells exposed to the KAO–ZEO extract proliferated well (their number noticeably increased with time) but at a slower rate compared to the control cells. The obtained results may therefore suggest ionic reactivity of KAO–ZEO formulation, causing slight changes in the ionic composition of the culture medium, which could have consequently resulted in an inferior cell proliferation potential over time [27].

In the next step of the study, the effect of ZEO and KAO–ZEO on collagen synthesis by BJ cells was determined (Figure 3c). The obtained results showed that ZEO at a concentration of 0.0156% and the KAO–ZEO extract significantly increased collagen synthesis (43.7 µg/mL and 41.6 µg/mL, respectively) compared to the control fibroblasts (23.9 µg/mL). Despite the lower proliferation potential of the fibroblasts exposed to the KAO–ZEO extract, they produced higher amounts of collagen compared to untreated cells, but similar amounts to those exposed to the ZEO (0.0156%). This may suggest that it was ZEO that was responsible for significant stimulation of collagen production by fibroblasts. ZEO can likely activate specific signaling pathways that lead to collagen production. If these pathways are highly activated, the resulting increase in collagen production can be significant, even with fewer cells. It should also be noted that cells during intensive synthesis of the proteins of extracellular matrix (ECM) often slow down their divisions to use the energy for this process, which is one of the most energy consuming processes in the cell [28,29]. It is worth to highlight that the ability of various essential oils (e.g., *Juniperus chinensis* L., and *Juniperus chinensis* var. *sargentii*) to boost skin elasticity by increasing expression of collagen and elastin is well known in the available literature [30]. Some authors suggested that increased collagen production upon exposure to essential oils or plant extracts results from enhanced transforming growth factor-β (TGF-β)/SMAD pathway that inhibits matrix metalloproteinase 1 (MMP-1, collagenase) expression [31,32]. TGF-β is a cytokine that binds to a TGF-β type II receptor (TβRII), leading to phosphorylation of a TGF-β type I receptor (TβRI) and activation of SMAD2 and SMAD3 transcription factors. Activated SMAD2 and SMAD3 combine with SMAD4 to form heteromeric SMAD complexes which are translocated into the cell nucleus, resulting in upregulation of collagen synthesis and downregulation of the expression of matrix metalloproteinases [33].

Kaolinite is used in various compositions due to its adsorptive, binding, and structural properties, especially in formulations related to environmental, pharmaceutical, and industrial applications [1]. Kaolinite, as part of the kaolin clay family, is commonly utilized in pharmaceuticals due to its excellent adsorption, low toxicity, and rheological properties. It serves multiple roles, including as a disintegrant, binder, and emulsifying agent, contributing to the stability and bioavailability of drugs. Kaolinite is particularly valued for its high purity and capacity for controlled drug release, making it ideal for formulations needing gradual release profiles [34]. Topically, it can protect the skin, and because it promotes blood clotting, it is effective in wound care products and hemostatic agents. A recent development even includes a kaolinite-based wound healing composite to help accelerate clotting and tissue repair [35]. Kaolin is one of the most important skin protectants, identified by the US food and drug administration, formulated into barrier creams used to prevent and treat mild irritant and allergic contact dermatitis originated at home and workplace with daily used agents such as weak solvents, detergents or even water by forming a thin impervious film [36,37]

Some studies focus on modifying kaolinite to enhance its drug-binding and release characteristics, such as coating it with cationic polymers to optimize drug delivery [38,39]. This functionalization allows it to act as a slow-release carrier, particularly in situations requiring extended therapeutic effects.

The composition of natural compounds is highly desirable for environmentally friendly applications. Kaolinite, valued for its non-toxic and inert qualities, is widely used as a base material in pharmaceutical and cosmetic products. When combined with natural additives, it contributes to cleaner formulations, reducing reliance on synthetic, potentially harmful ingredients. KAO–ZEO is the first documented formulation of kaolinite combined with Ziziphora essential oil, showing significant antimicrobial activity and the ability to stimulate collagen production in fibroblasts. This makes it a promising new treatment option for skin infections.

According to the European Wound Management Association (EWMA) guidelines, proper wound cleaning and debridement are foundational to care. Removing necrotic tissue and contaminants helps control the infection and promote healing. Wound dressings with antimicrobial properties (e.g., silver-containing dressings) are usually used to manage superficial infections or as adjuncts to systemic therapy when needed. These interventions aim to reduce microbial burden, enhance the wound-healing environment, and minimize complications [40]. In our opinion, integrating emerging technologies, such as kaolinite combined with Ziziphora essential oil, may provide additional benefits and synergize with current care standards by improving antimicrobial efficacy and wound healing outcomes. Kaolinite acts as an adsorbent that can trap bacterial toxins, while Ziziphora essential oil has demonstrated broad-spectrum antimicrobial activity. This combination aligns with the goal of preventing and treating infections in wound care.

In the next stage, conducting in vivo studies using animal models, such as rodents with simulated wound types (e.g., burns, lacerations, or diabetic wounds), would be highly beneficial. These studies should evaluate anti-inflammatory potential, wound closure rates, histology (e.g., granulation tissue, re-epithelialization), and infection control. Additionally, it is crucial to monitor for local or systemic adverse effects, such as inflammation or delayed healing.

## 3. Materials and Methods

### 3.1. Micronized Kaolinite Clay Powder Preparation

The source of natural kaolinite was obtained from the Alekseevskoye clay mineral deposit of the Akmola region in the Republic of Kazakhstan located between the villages of Vasilkovka and Birlistik, 27 km northwest of Kokshetau [10]. The method of obtaining the final product involves the sevenfold suspension (levigation) of the clay in purified water, fractionation, followed by drying and micronization of the raw material. After the purification of the clay minerals using special technology, they were tested for soluble barium salts, arsenic impurities, and heavy metals, in accordance with the requirements of the State Pharmacopoeia of the Republic of Kazakhstan. Laboratory and pilot-industrial protocols for obtaining purified kaolinite substance (CLEANED CLAY) and micronized kaolinite substance (kaolin light) were developed. A product standard for “CLEANED CLAY” [41] was obtained (introduced for the first time), and a patent application was submitted for the utility model “Method for obtaining micronized kaolinite clay powder for use in pharmacy, medicine, veterinary medicine, and cosmetology”. The technology for obtaining kaolinite substance was developed under laboratory conditions at the Practical Skills Center of Asfendiyarov Kazakh National Medical University. The technology was transferred, and the optimal technological parameters were fine-tuned in pilot-industrial conditions at the production facility of “FitOleum” LLP in Issyk. A pilot-industrial protocol was developed, and an application for patent for a utility model was submitted (patent for utility model RK №1787, IPC A61L 33/00, CO4B 3/04, published 15 November 2016).

### 3.2. Plant Material

The aerial parts of *Ziziphora bungeana* Lam. were collected in the summer of 2023 in the flowering stage in the Turkestan region of the Republic of Kazakhstan and identified by the Institute of Botany and Phytointroduction, Science Committee, Ministry of Education and Science of the Republic of Kazakhstan. A voucher sample №01-05/324 from 06.09.2023 has been deposited in the herbarium of the Institute of Botany and Phytointroduction, Almaty, Republic of Kazakhstan. A total of 50 g of the plant material of *Ziziphora bungeana* Juz. was crushed, ground, and subjected to 3 h hydro-distillation in Deryng-type apparatus with 400 mL of water to obtain essential oil (EO). A total of 380 g of raw material was hydro-distilled. The oil was dried over anhydrous sodium sulfate and stored in sealed amber vials at 4 °C prior to analysis.

### 3.3. Gel Ingredients

The following chemicals were used as gel ingredients: Xanthan gum, polyglyceryl 4-caprate and Aristoflex AVC (ammonium acryldimethyltaurate/N-vinyl pyrrolidone copolymer) were purchased from GC S.r.l. (Milano, Italy), Neusilin UFL-2 (syntethic amorphous magnesium aluminosilicate) was purchased from Fuji Chemical Industries (Osaka, Japan), distilled water and anhydrous glycerin were purchased from Avantor Performance Materials (Gliwice, Poland).

### 3.4. GC–MS

GC–MS analysis was carried out using a Shimadzu GC-2010 Plus coupled with a Shimadzu QP2010 Ultra mass spectrometer (Kyoto, Japan). A fused silica capillary column ZB-5 MS (30 m, 0.25 mm i.d.) with a film thickness of 0.25 mm (Phenomenex, Torrance, CA, USA) was used. The oven temperature program was started at 50 °C, held for 3 min, then increased at the rate of 8–250 °C/min, and held for a further 2 min. The MS was operated in EI mode; the scan range was 40–500 amu, the ionization energy was 70 eV, and the scan rate was 0.20 s per scan. The injector (250 °C), interface (250 °C), and ion source (220 °C) temperatures were set. Split injection was performed with a split ratio of 1:20. Helium was the carrier gas at a 1.0 mL/min flow rate. *Z. bungeana* EOs samples were prepared by diluting 1 µL of EO in 999 µL of hexane. The retention indices (RI) were determined in relation to a homologous series of n-alkanes (C8–C24) under the same operating conditions. Constituents of the studied essential oils were identified by comparison of their mass spectra and retention indices with computer-supported spectral libraries (Mass Finder 2.1 and NIST database).

### 3.5. Kaolin–Ziziphora Essential Oil (KAO–ZEO) Therapeutic Mix Preparation

White micronized kaolinite clay powder (KAO) and Ziziphora essential oil (ZEO) were used to produce gel formulation. Aside from active ingredients, following excipients were utilized: ammonium acryldimethyltaurate/N-vinyl pyrrolidone copolymer as thickening agent, xanthan gum as texturizer, synthetic amorphous magnesium aluminometasilicate as dispersing agent, glycerin as wetting agent, polyglyceryl 4-caprate as solubilizer. Water and glycerin were mixed in a beaker, ammonium acryldimethyltaurate/N-vinyl pyrrolidone copolymer and xanthan gum were dispersed and mixed with a MICROSTAR 7.5 control overhead stirrer (IKA, Staufen, Germany) equipped with a dissolver stirrer at a speed of 200 rpm until a clear gel was formed. Further, polyglyceryl 4-caprate and synthetic amorphous magnesium aluminosilicate were dispersed at a speed of 400 rpm. Lastly, white micronized kaolinite was added in portions and stirred at 1000 rpm and finally Ziziphora essential oil was added to the formulation with gentle stirring at 100 rpm. Formulation content is summarized in Table 4.

In addition to KAO–ZEO gel, two reference formulations were prepared in order to assess separate activity of micronized kaolin clay (KAO-mix) and Ziziphora essential oil (ZEO-mix) in gel matrix. The third formulation contained only excipients with no active ingredients. The subtracted mass of micronized kaolin clay or Ziziphora essential oil were replaced with water in all three reference formulations.

### 3.6. Formulation Assessment

Gel viscosity was assessed using a vibrational viscometer SV-10 A&D (Tokyo, Japan) equipped with a water bath circulator LWT 2/200WSL. A plastic cuvette was placed in a vessel connected to a circulator and further filled with 10 mL of gel formulation. The sample was stabilized for 10 min at the temperature of 25 °C before measurements. Viscosity was recorded for 3 separate samples and averaged. The gel density was assessed by use of EasyDens portable density meter (Anton Paar, Graz, Austria). Briefly, a sample of 1 mL was injected into the apparatus and the measuring cell was cleared with distilled water following the measurement. Five parallel samples were investigated.

Mixture pH was measured by use of pH-metr PH5, Dostmann (Wertheim-Reicholzheim, Germany) using 5 parallel samples. Briefly, a sample of 1 g of gel was weighted and transferred into a beaker and diluted with distilled water in order to obtain a 10% aqueous solution.

### 3.7. Antimicrobial Activity Testing

The ZEO dissolved in dimethylosulfoxide (DMSO) and micronized kaolinite clay powder (KAO) dissolved in water were screened for antibacterial and antifungal activities by micro-dilution broth method according to both the European Committee on Antimicrobial Susceptibility Testing (EUCAST) (www.eucast.org, accessed on 15 November 2024) using Mueller–Hinton broth (MH) and MH supplemented with 5% of hemolyzed horse blood for non-fastidious and fastidious bacteria, respectively, and RPMI with MOPS for the growth of fungi, as we described elsewhere [42]. The Minimal Inhibitory Concentration (MIC) of the tested extracts was evaluated for the wide panel of the reference microorganisms from, including Gram-negative bacteria (*Escherichia coli* ATCC 25922, *Klebsiella pneumoniae* ATCC 13883, *Proteus mirabilis* ATCC 12453, *Salmonella typhimurium* ATCC 14028, *Pseudomonas aeruginosa* ATCC 9027, *Moraxella catarrhalis* ATCC 25238), Gram-positive bacteria (*Staphylococcus aureus* ATCC 25923, *Staphylococcus aureus* ATCC 43300, *Staphylococcus epidermidis* ATCC 12228, *Micrococcus luteus* ATCC 10240, *Enterococcus faecalis* ATCC 29212, *Streptococcus pyogenes* ATCC 10876, *Streptococcus pneumoniae* ATCC 49619), and fungi (*Candida albicans* ATCC 10231, *Candida parapsilosis* ATCC 22019, *Candida glabrata* ATCC 90030, *Candida auris* CDC B11903, *Candida krusei* ATCC 14243). The sterile 96-well polystyrene microtitrate plates (Nunc, Roskilde, Denmark) were prepared by dispensing 100 µL of appropriate dilution of the tested extracts in broth medium per well by serial two-fold dilutions in order to obtain final concentrations of the tested ZEO and KAO ranged from 10 to 0.0195 mg/mL and KAO–ZEO from 100 to 0.78 mg/mL The inocula were prepared with fresh microbial cultures in sterile 0.85% NaCl to match the turbidity of 0.5 McFarland standard were added to wells to obtain final density of 5 × 10^5^ CFU/mL for bacteria and 5 × 10^4^ CFU/mL for yeasts; CFU—colony forming units. After incubation (35 °C for 24 h), the MICs were assessed visually as the lowest concentration of the extracts showing complete growth inhibition of the reference microbial strains. An appropriate DMSO control (at a final concentration of 10%), a positive control (containing inoculum without the tested derivatives), and negative control (containing the tested derivatives without inoculum) were included on each microplate.

Minimal bactericidal concentration (MBC) or minimal fungicidal concentration (MFC) was obtained by culture of 5 µL from each well that showed through growth inhibition, from the last positive one, and from the growth control onto recommended agar plates.

The MBC/MFC was defined as the lowest concentration of extract without the growth of microorganisms. The MBC/MIC ratios were calculated to determine the bactericidal or bacteriostatic effect of the assayed extract. Vancomycin, clarithromycin, ciprofloxacin, and nystatin were used as the reference drugs appropriate for different group of microorganisms. The experiments were repeated in triplicate. Representative data are presented.

### 3.8. Evaluation of the Cellular Response to ZEO and KAO–ZEO In Vitro

#### 3.8.1. Cell Culture Experiments

Normal human skin fibroblasts (BJ), obtained from American Type Culture Collection (ATCC-LGC Standards, Teddington, UK), were cultured in Eagle’s Minimum Essential Medium (EMEM, ATCC-LGC Standards, Teddington, UK) containing 10% fetal bovine serum (Pan-Biotech GmbH, Aidenbach, Bavaria, Germany) and 1% penicillin–streptomycin solution (Sigma-Aldrich Chemicals, Warsaw, Poland). The cells were maintained at 37 °C in a humidified atmosphere with 5% CO_2_.

#### 3.8.2. Screening Cytotoxicity Test

To screen the cytotoxicity of ZEO, different concentrations ranging from 0.0039% to 0.2500% were tested and prepared by serial two-fold dilutions in the complete culture medium. In turn, a cytotoxicity assessment of KAO–ZEO was performed according to the ISO 10993-5 standard [43] procedure by an indirect method, using its 24 h extract prepared in accordance with the ISO 10993-12 standard [44] procedure (100 mg of the sample were soaked in 1 mL of complete culture medium for 24 h at 37 °C). First, 100 μL of BJ cells’ suspension at a concentration of 2 × 10^5^/mL were added into wells of 96-well plates. After a 24 h incubation, the culture medium was discarded, and the cells were exposed to 100 μL of the various concentrations of ZEO and KAO–ZEO extract. BJ cells maintained in culture medium without the ZEO and KAO–ZEO served as a control. The cells were incubated for 24 h, after which cytotoxicity was assessed by the MTT assay (Sigma-Aldrich Chemicals, Warsaw, Poland). The results were expressed as a percentage of the absorbance value obtained from the control cells, which represented 100% viability. The highest concentration of ZEO that did not decrease cell viability below 90% was selected to perform cell proliferation and collagen synthesis assessment.

#### 3.8.3. Cell Proliferation Assessment

A total of 1 × 10^4^ of BJ cells were seeded into the wells of 96-well plates. After a 24 h incubation, the culture medium was discarded, and the cells were exposed to 100 μL of the highest non-toxic concentration of ZEO (0.0156%) and KAO–ZEO extract which was prepared in accordance with the ISO 10993-12 standard procedure (100 mg of the sample were soaked in 1 mL of complete culture medium for 24 h at 37 °C). The BJ cells cultured in culture medium without the ZEO and KAO–ZEO served as a control. On the third day of the experiment, half of the culture medium was refreshed. The number of cells was assessed after 1, 2, 3, and 4 days of culture using the Cell Counting Kit-8 (WST-8 assay, Sigma-Aldrich Chemicals, Warsaw, Poland) according to the manufacturer’s instructions.

#### 3.8.4. Evaluation of Collagen Synthesis

To evaluate collagen synthesis, the BJ cells were cultured as described in Section 3.8.3. After seven days of culture, collagen synthesis was assessed calorimetrically using the Sirius Red Total Collagen Detection Assay Plate Kit (Chondrex, Woodinville, WA, USA) according to the manufacturer’s protocol.

#### 3.8.5. Statistical Analysis

All experiments were conducted in triplicate. For the cell culture experiments, statistically significant differences between control cells maintained in the culture medium and cells treated with ZEO and KAO–ZEO were determined using One-way ANOVA with a post hoc Dunnett’s test, with the significance set at *p* < 0.05 (GraphPad Prism 8.0.0 Software, San Diego, CA, USA).

## 4. Conclusions

KAO–ZEO shows potential as an innovative formulation combining kaolinite, a natural geomaterial, with Ziziphora essential oil. Together, these natural compounds may offer promising new approaches for addressing global health challenges, including skin infections caused by antibiotic-resistant bacteria. Topically, it can protect the skin, and because it stimulates collagen production using fibroblasts and shows antimicrobial activity, it can be considered as a novel treatment for skin infection.

## Figures and Tables

**Table 1:** The chemical composition of Ziziphora EO (ZEO).

No	RT	RI_exp_	RI_db_	Area (%)	Name
1	7.821	931	936	0.19	α-pinene
2	9.094	971	978	0.09	β-pinene
3	9.249	976	973	0.25	Sabinene
4	9.636	988	987	0.16	β-myrcene
5	9.960	998	979	0.11	3-octanol
6	10.955	1028	1018	0.73	Limonene
7	11.040	1031	1024	0.15	1,8-cineol
8	13.344	1100	1082	0.11	Linalool
9	14.977	1152	1150	0.58	Borneol
10	15.118	1156	1136	0.12	Menthone
11	15.408	1165	1146	10.28	*iso*-menthone
12	15.734	1175	1179	1.26	Isopulegone
13	16.321	1194	1176	1.06	Isomenthol
14	17.160	1221	1220	0.36	p-isopropylbenzaldehyd
15	17.734	1240	1216	72.98	Pulegone
16	19.514	1300	1278	0.13	Carvacrol
17	20.665	1340	1318	6.96	Piperitenone
18	22.937	1421	1421	0.29	Caryophyllene

**Table 2:** Antimicrobial activity of Ziziphora Essential oil (ZEO), and micronized kaolinite clay powder (KAO).

Chemicals Microorganism	ZEO		KAO	
	MIC (mg/mL)	MBC (mg/mL)	MIC (mg/mL)	MBC (mg/mL)
**Gram-positive bacteria**				
*S. aureus* ATCC 25923	5	10	10	>20
*S. aureus* ATCC BA1707	5	10	10	>20
*S. epidermidis* ATCC 12228	5	10	10	>20
*M. luteus* ATCC 10240	2.5	5	5	20
*B. cereus* ATCC 10876	5	20	10	>20
*E. faecalis* ATCC 29212	5	20	10	>20
*S. pneumoniae* ATCC 49619	1.25	1.25	5	5
*S. pyogenes* ATCC 19615	2.5	2.5	5	5
**Gram-negative bacteria**				
*S. typhimurium* ATCC 14028	5	10	10	>20
*E. coli* ATCC 25922	2.5	2.5	10	20
*P. mirabilis* ATCC 12453	5	5	10	>20
*K. pneumoniae* ATCC 13883	5	10	10	>20
*P. aeruginosa* ATCC 9027	5	5	10	20
*M. catarrhalis* ATCC 25238	0.313	0.313	2.5	10
**Yeasts**				
*C. glabrata* ATCC 90030	2.5	5	10	10
*C. albicans* ATCC 102231	1.25	2.5	5	10
*C. parapsilosis* ATCC 22019	2.5	5	5	10
*C. krusei* ATCC 14243	1.25	2.5	5	10
*C. auris* CDC B11903	1.25	2.5	5	10

**Table 3:** MIC values (mg/mL) of different variants of mixture.

Chemicals Microorganism	KAO–ZEO Mixture	ZEO Mixture	KAO Mixture	Excipients
**Gram-positive bacteria**				
*S. aureus* ATCC 25923	25	>100	25	>100
*S. aureus* ATCC BA1707	25	>100	25	>100
*S. aureus* ATCC 43300	0.32	>100	0.32	>100
*S. epidermidis* ATCC 12228	0.16	50	25	>100
*M. luteus* ATCC 10240	0.32	0.32	0.32	>100
*E. faecalis* ATCC 29212	0.32	>100	0.32	>100
*S. pyogenes* ATCC 19615	>100	>100	>100	>100
**Gram-negative bacteria**				
*E. coli* ATCC 25922	25	>100	25	>100
*P. aeruginosa* ATCC 9027	50	>100	50	>100
**Yeasts**				
*C. glabrata* ATCC 90030	25	>100	100	>100
*C. albicans* ATCC 102231	0.32	>100	100	>100
*C. parapsilosis* ATCC 22019	0.08	50	100	>100

**Table 4:** Ingredients of gel formulation containing micronized kaolinite and Ziziphora essential oil (KAO–ZEO).

Ingredient	Content (%; *w*/*w*)
white micronized kaolinite	30
Zizphora essential oil	0.25
ammonium acryldimethyltaurate/N-vinyl pyrrolidone copolymer	0.5
xanthan gum	0.5
polyglyceryl 4-caprate	0.5
synthetic amorphous magnesium aluminosilicate	0.6
glycerin	10.0
water	57.65

## Data Availability

Data is contained within the article.
